# Use of different subjective health indicators to assess health inequalities in an urban immigrant population in north-western Italy: a cross-sectional study

**DOI:** 10.1186/1471-2458-13-1006

**Published:** 2013-10-25

**Authors:** Alexander Domnich, Daniela Amicizia, Donatella Panatto, Alessio Signori, Valentina Perelli, Sergio Adamoli, Edoardo Berti Riboli, Roberto Gasparini

**Affiliations:** 1 Department of Health Sciences, University of Genoa, via Pastore, 1-16132, Genoa, Italy; 2 Non-Profit Organization “Medici in Africa”, largo Rosanna Benzi, 8-16132 Genoa, Italy

**Keywords:** Italy, Immigrants, Subjective health indicators, Health-related quality of life (HRQoL), Self-rated health (SRH), Health inequalities

## Abstract

**Background:**

Despite the steady growth of the immigrant population in Italy, data on the health status of immigrants are scarce. Our main goals were to measure Health-Related Quality of Life (HRQoL), Self-Rated Health (SRH) and morbidity among immigrants in Genoa. We aimed to assess the relative contribution of some social, structural and behavioral determinants to “within-group” health disparities.

**Methods:**

We enrolled 502 subjects by means of snowball sampling. The SF-12 questionnaire, integrated with socio-demographic and health-related items, was used. Multivariate logistic and Poisson regression models were applied in order to identify characteristics associated with poor SRH, lower SF-12 scores and prevalence of self-reported morbidities.

**Results:**

Subjects showed relatively moderate levels of HRQoL (median physical and mental scores of 51.6 and 47.3, respectively) and about 15% of them rated their health as fair or poor. Lower scores in the physical dimension of HRQoL were associated with the presence of morbidities and immigration for work and religious reasons, while those who had migrated for religious and family reasons displayed a lower probability of lower scores in the mental dimension of HRQoL. Poor SRH was associated with female gender, overweight/obesity and presence of morbidities. Moreover, compared with immigrants from countries with a low human development index, immigrants from highly developed societies showed significantly lower odds of reporting poor SRH. About one-third of respondents reported at least one medical condition, while the prevalence of multi-morbidity was 10%. Females, over 45-year-olds, overweight and long-term immigrants had a higher prevalence of medical conditions.

**Conclusions:**

Our study confirms the presence of health inequalities within a heterogeneous immigrant population. HRQoL, SRH and morbidity are valid, relatively rapid and cheap tools for measuring health inequalities, though they do so in different ways. These indicators should be used with caution and, if possible, simultaneously, as they could help to identify and to monitor more vulnerable subjects among immigrants.

## Background

Immigration is a relatively recent phenomenon in Italy, a country previously associated with emigration. Indeed, the net migration balance became positive only in the last quarter of the XX^th^ century [1]. Genoa, like other Italian cities, has experienced a significant increase in immigration from other countries, especially from Latin America. According to the Italian National Institute of Statistics (ISTAT) foreigners (about 50,000) account for 8.3% of the city’s total population; this is higher than the regional (7.8%) and national (7.5%) averages [2]. The top ten foreign communities in Genoa are Ecuadorians, Albanians, Moroccans, Romanians, Peruvians, Chinese, Ukrainians, Senegalese, Sri Lankans and Bangladeshis [2].

It is well known that migration has a significant impact on the physical, mental and emotional health and well-being of migrants [3]. The complex determinants of migrants’ health are interrelated and have not yet been fully understood. The health of immigrants is influenced by three main groups of factors: pre-migration factors (such as socio-economic development and environmental aspects of the country of origin), the travel or migration process itself (for example, psycho-social burdens, stress, hunger, separation from the family, etc.) and post-migration factors (such as community support, racism, access to healthcare services, etc.) [4].

Most studies of migrants’ health, especially cross-sectional studies, have compared the characteristics of a cohort of immigrants with those of a demographically similar cohort of natives in the host country, and have investigated health disparities (or inequalities) between these two cohorts [5-10]. Some of these studies have found that, on arrival in a host country, immigrants are in better health than native-born individuals, but that this initial health advantage diminishes over the time of residence. This phenomenon has been dubbed the “healthy immigrant” effect (HIE) and may be explained by several theories (immigrant self-selection prior to migration, “cultural buffering”, health screening processes and others) [5-8,11-16]. By contrast, other studies have recorded poor health among immigrants, a feature that has been termed the “sick immigrant” paradigm [11]. However, as immigrants and ethnic minorities constitute a very heterogeneous group [17], it is worth analyzing health inequalities within the migrant population.

Apart from the health inequalities linked to genetic and biological aspects, social variables are an important source of disparities in health. Social variables may be further divided into socio-structural, behavioral and psychosocial factors [18]. Several socio-structural factors have been linked to health, including age [19,20], gender [18,21], educational level [22-24], marital status [25,26] and several others. Analysis of structural factors in immigrant populations should also consider the power dimensions of race, gender and immigrant status hierarchies, in order to understand how these shape health disparities [27]. Behavioral or lifestyle determinants, such as smoking, alcohol consumption and being overweight, are strictly connected to health and illness [18] as well as to health-related quality of life (HRQoL) [28]; indeed, 60% of deaths are attributable to unhealthy (“westernized”) lifestyle patterns [29]. Foreign-born individuals have been seen to display a higher risk of smoking [30], drinking [31] and obesity [32] after years of residence in a host country than initially (soon after arriving), which seems to be due to an acculturation process. Indeed, the model of lifestyle acculturation implies a decline in health over the time of residence [33]. This model is somewhat in contrast with the so-called “cultural buffering” mechanism, according to which immigrants from less modern societies tend to maintain their original cultural and lifestyle practices and expectations [34]. Psycho-social factors are different from socio-structural ones, as they occur at the individual, subjective level and include, for example, critical life events and chronic stressors [18].

Objective methods of measuring health are often too expensive to implement on a sufficiently large sample of subjects, especially in the case of immigrants, who are difficult to reach. Therefore, self-assessment techniques, such as Self-Rated Health (SRH) and HRQoL, have been developed as a cheaper means of measuring health disparities [35]. SRH is an indicator of general health status, and frequently consists of only one Likert-based question [36-38]. Despite its simplicity, SRH has been shown to display a high level of correlation with objective health measures such as mortality, morbidity and health control strategies [37]. By contrast, HRQoL is a multidimensional index referring to the physical, psychological and social domains of health and well-being, which are influenced by personal experiences, beliefs, expectations and perceptions [39]. These two indexes are often used interchangeably, not least because the SRH concept is often included in that of HRQoL. However, HRQoL is more widely applied, owing to its multidimensionality [38]. Another widely used indicator is self-reported morbidity, which is often employed in population-based studies when individual data are not available; this has also been used to examine equity issues [40].

Subjective health indicators have often been used in migrant studies in Europe. In one such investigation, Nesterko et al. [41] noted an association between the country of origin and the physical dimension of HRQoL among migrants to Germany; the authors therefore claimed that there is a need to focus attention on migrant-related factors rather than simply comparing migrants and natives. In a Czech study [36] the probability of reporting poor SRH was found to be higher among women and older immigrants, while educational level and legal status were not able to predict SRH. Finally, a Swedish cross-sectional study [42] found a negative impact of short time of residence on poor perceived health.

As mentioned above, health inequalities between population groups result from numerous factors. Recently, the concept of total health inequalities has been introduced, which is the sum of “between-group” and “within-group” inequalities; research has mainly focused on the “between-group” component [43], i.e. immigrants versus natives. However, as migration itself is a strong determinant of health, the causes of health disparities can vary among migrants and natives. Analyzing “within-group” differences may improve our understanding of the nature of health disparities and thus contribute to eliminating them [44]. Indeed, immigrant populations comprise different categories: economic migrants, international students, migrants for family reasons and so on, and each group faces different health challenges [45]. Another factor that can affect the health outcomes of migrants in a host country is their place of birth. Indeed, the health features of migrants in the pre-departure phase can differ substantially, reflecting disparities in the determinants of health not only among individuals but also at the societal level [46]. The effect of the place of birth on migrants’ health is expected to be linked to the average level of health in the country of origin, socialization of health behavior during migrants’ childhood or level of political suppression in the source countries [47]. European research on this issue has documented mixed patterns of the influence of birth place on migrants’ health, and is far from conclusive. For instance, in Sweden, immigrants from Southern Europe displayed poorer health and a higher risk of cardiovascular disease than immigrants from other regions [48]. Moroccan immigrants in the Netherlands displayed lower mortality and cardiovascular risks than Surinamese and Antillean immigrants [49,50]. German and French migrants to Switzerland enjoyed better health than those from Italy, ex-Yugoslavia, Portugal, Spain and Turkey [51].

In spite of the rapid increase in immigration in Italy, data on the health status of immigrants are scarce. The main goal of the present study was to quantify the health status of foreign-born residents in Genoa by measuring HRQoL, SRH and self-reported morbidities. On the basis of research conducted in various socio-economic and geographical settings and among subjects with different migration histories [36,41,42,48-51], we hypothesized the presence of health disparities within the immigrant community in Genoa. Our research question concerned the relative contribution of some socio-structural, behavioral and pre-departure determinants of inequalities in health. To this end, we applied regression-based models of individual health, which can be used to examine health disparities [52]. We also aimed to assess the ability of each subjective health indicator to assess health inequalities within the study population.

## Methods

### Data collection

Enrollment in this cross-sectional study took place between January and August 2012. To be eligible for inclusion, subjects had to be born abroad, not have Italian nationality, have a sufficient knowledge of Italian to fill in the questionnaire, be ≥ 18 years old and live in the city of Genoa.

Owing to the quantitative and qualitative heterogeneity of foreign communities [2] and the absence of a survey frame, snowball sampling was used. For this reason, the results cannot be generalized to the entire immigrant population. Initial seeds were recruited at parish centers, student hostels, cultural and social promotion associations, and during communities’ cultural events.

All participants were contacted personally and were informed as to the objectives of the survey and the voluntary nature of participation. Anonymous questionnaires were distributed only to those who had agreed to participate in the survey. There was no risk of any single participant being identified. The Ethics Committee of S. Martino Hospital (Genoa, Italy) approved the study protocol (N^o^ 20/2011).

### Questionnaire

We used a questionnaire that consisted of the SF-12 Short Form Health Survey (SF-12), version 1 [53-55], integrated with items regarding socio-demographic characteristics and immigration background (gender, age, birth country, marital status, level of education, reason for migration, year of arrival in Italy), some common risk factors (current smoking status, alcohol intake, height, weight) and self-reported medical conditions.

The SF-12, which is the abbreviated version of the SF-36, is particularly suitable and advantageous for use in large-scale (n = 500+) health measurement and in situations in which there is a need to achieve summary information on physical and mental health status. The SF-12 comprises twelve five-point Likert-based questions, which cover eight health dimensions: physical and social functioning, role limitations due to physical health and role limitations due to emotional problems, mental health, vitality, bodily pain and general health. The SF-12 yields two composite scores, the Physical Component Summary (PCS-12) and the Mental Component Summary (MCS-12); a higher score corresponds to a better health status. The SF-12v1 has been validated for use in the Italian population [53-55]. The single Likert-based item on SRH was the question “Would you say your health is excellent, very good, good, fair, or poor?” [36-38].

All study participants were asked about their present smoking status (“Do you currently smoke?”). Questions on alcohol consumption concerned the frequency, quantity and type (beer, wine or spirits); intake was then converted into standard units (12.5 g of ethanol/drink) and dichotomized into non-drinking/light drinking (≤ 1 drink per day) and moderate/heavy drinking (> 1 drink per day) [56]. Participants were also asked to report any health problems and their height and weight, in order to calculate Body Mass Index (BMI).

Questionnaires were self-completed by participants. All questionnaires were checked on the basis of quality control; only fully completed questionnaires were considered, as missing responses to any of the twelve SF-12 items makes it impossible to calculate both summary scores.

### Socio-demographic characteristics

Age was categorized according to the following age-classes: 18–24, 25–34, 35–44, 45–54, 55+. Marital status was dichotomized into (1) single never married, divorced/separated, widowed and (2) married/cohabiting. Educational level was defined as “middle school or lower” and “high school or higher”. Reasons for migration were classified as: study, work, family and religious reasons. Immigrants for religious reasons were defined as those whose first residence permit had been issued for religious reasons. Participants were divided into 2 groups on the basis of the time spent in Italy: recent (< 10 years) and long-term (≥ 10 years). This division was based on previous research on social determinants of health in an immigrant population [57].

### Country of birth

As a pre-migration factor, the country of origin was classified in accordance with the Human Development Index (HDI) promoted by the United Nations (UN) [58]. The HDI is a summary composite index of human development that measures a country’s average achievements in three basic dimensions of human development: health (measured by life expectancy at birth), education (measured by mean years of schooling and expected years of schooling) and standard of living (measured by per capita Gross National Income [GNI]). The 2011 HDI classification uses a relative approach based on quartiles (from “very high” to “low”) [58].

### Statistical analysis

SF-12 scoring was carried out by means of the algorithms described by Apolone et al. [53]; to obtain final PCS-12 and MCS-12 scores, physical and mental weights corresponding to a response choice were summed and then standardized by adding a constant (different for the two summary scores) to the sum of physical or mental weights [53].

Quantitative variables (SF-12 scores) were expressed as means and standard deviations (SD) or medians and interquartile ranges (IQRs), while qualitative variables were expressed as frequencies and percentages.

As the distribution of SF-12 scores was asymmetric, the Mann–Whitney U test was used to evaluate differences in quantitative variables between two groups (sex, marital status, schooling, duration of residence, current smoking, alcohol consumption, presence/absence of overweight/obesity and medical conditions), while the non-parametric Kruskal-Wallis test was used for three or more groups (age-class, reason for migration and HDI quartile of birth country). The chi-square test was used to compare categorical data, i.e. percentages of subjects who reported poor/fair SRH and those who reported good/very good/excellent SRH by sex, age-class, marital status, schooling, HDI of birth country, reason for migration, duration of residence, smoking, alcohol consumption, BMI, and presence/absence of medical conditions. Student’s t test was used to estimate differences in current age and age on arrival in Italy between genders. Spearman’s correlation coefficient was used to measure the correlation between SF-12 subscales, SRH and count of medical conditions.

Multivariate regression models (logistic for SRH and SF-12 scores and Poisson for medical conditions) were utilized to determine whether outcomes were associated with demographic and lifestyle variables. Those characteristics with a p-value lower than 0.15 on univariate analysis were considered in a multivariate model. A stepwise forward approach was used to select the variables for the final multivariate model, with a p-value of exclusion set to 0.10. The independent variables considered in regression models were: gender, age-class, time of residence, HDI of birth country, reason for migration, marital status, level of education, smoking status at the time of the survey, alcohol consumption in standard drinks per day and BMI. Measures of relative effect were expressed as odds ratios (ORs) for logistic regression and prevalence ratios (PRs) for Poisson regression, with 95% confidence intervals (CIs). The level of significance adopted was p < 0.05. All data were analyzed by means of the R stats package, version 2.15.1 [59].

## Results

### Study population

Initially, 614 subjects were enrolled. Of these, 19 were unable to fill in the questionnaire because of language difficulties, 9 were aged less than 18 years, and 4 had been born in Italy. Another 58 subjects refused to participate because of lack of interest in the topic; the majority of these were females (64%) and subjects from Eastern Asia (42%). We also excluded 22 questionnaires from further analysis on the basis of quality control (not fully completed). Therefore, 502 questionnaires were analyzed.

### Descriptive statistics

The subjects enrolled were from 49 different countries (Table 1). Their mean age was 35.5 (SD 12.7) years (M: 32.4 [SD 11.4] years; F: 39.1 [SD 13.2] years, t = 6.10, p < 0.001) and about 70% of the sample fell into the prime working age (25–55 years). Their mean age on arrival in Italy was 26.6 (SD 10.3) years and was significantly (t = 4.30, p < 0.001) lower among men than among women (M: 24.8 [SD 9.4] years; F: 28.7 [SD 10.9] years). About three-quarters of subjects had arrived in Italy at the age of 15–35 years. Table 2 reports the socio-demographic characteristics of the participants and three important health risk behaviors.

**Table 1:** Distribution of participants according to birth country

**HDI category (HDI value)**[58]	**Number (%)**	**Countries**
Low (0.510-0.286)	121 (24.1)	Angola, Bangladesh, Cameroon, Eritrea, Ethiopia, Nigeria, Pakistan, Senegal, Somalia*, Sudan, Tanzania, Togo
Medium (0.698-0.522)	120 (23.9)	Algeria, Bolivia, Cape Verde, China, Congo-Brazzaville, Dominican Republic, Egypt, Gabon, India, Jordan, Moldova, Morocco, Occupied Palestinian Territory, Philippines, Sri Lanka
High (0.783-0.698)	219 (43.6)	Albania, Belarus, Colombia, Cuba, Ecuador, Iran, Lebanon, Mexico, Peru, Romania, Russia, Tunisia, Ukraine, Uruguay, Venezuela
Very high (0.943-0.793)	42 (8.4)	Argentina, Chile, France, Greece, Israel, Poland, Spain

*: No HDI data on Somalia are provided in the 2011 Report; Somalia was last included in the HDI ranking in 2001 [58].

**Table 2:** Socio-demographic characteristics and lifestyle risk factors of participants

**Variable**	**Level**	**Number**	**%**
Sex	Male	275	54.8
	Female	227	45.2
Age-class at survey, years	18-24	105	20.9
	25-34	174	34.7
	35-44	105	20.9
	45-54	70	13.9
	55+	48	9.6
Marital status	Never married/divorced/widowed	279	55.6
	Married/cohabiting	223	44.4
Schooling	Middle school or lower	149	29.7
	High school or higher	353	70.3
Reason for migration	Study	127	25.3
	Work	257	51.2
	Family	98	19.5
	Religious	20	4.0
Duration of residence, years	< 10	296	59.0
	≥ 10	206	41.0
Current smoking status	Non-smoker	398	79.3
	Smoker	104	20.7
Alcohol consumption, drinks per day	≤ 1	461	91.8
	> 1	41	8.2
BMI, kg/m^2^	< 25	337	67.1
	≥ 25	165	32.9

The majority of participants rated their health as excellent, very good or good; about 15%, however, rated their health as poor or fair (hereinafter “poor”) (Table 3). Overall, PCS-12 scores ranged from 18.4 to 67.7 and MCS-12 scores from 17.7 to 66.6. The distributions of both SF-12 scores were negatively asymmetric, with medians greater than means (Table 3) and skewness coefficients of -0.80 and -0.34 for PCS-12 and MCS-12, respectively.

**Table 3:** Self-rated health and median SF-12 scores reported by subjects (n = 502)

**SRH, N (%)**					**PCS-12 score**		**MCS-12 score**	
**Poor**	**Fair**	**Good**	**Very good**	**Excellent**	**Mean (SD)**	**Median (IQR)**	**Mean (SD)**	**Median (IQR)**
11 (2.2)	66 (13.1)	216 (43.0)	146 (29.1)	63 (12.6)	49.7 (8.5)	51.6 (43.7-55.9)	46.2 (10.1)	47.3 (38.4-54.0)

Table 4 shows the percentages of subjects with poor SRH and the median physical and mental scores according to the main variables considered.

**Table 4:** Percentage of subjects (n = 502) who reported poor or fair health status and median SF-12 scores according to the main variables considered

**Variable**	**Level**	**N/total (%) of poor SRH**	**χ**^**2 **^**(p)**	**PCS-12 median score (IQR)**	**Non parametric test (p)**	**MCS-12 median score (IQR)**	**Non parametric test (p)**
Sex	Male	25/275 (9.1)	17.23 (<0.001)	52.7 (46.8-56.6)	3.88^a^ (<0.001)	47.5 (38.6-54.1)	0.75^a^ (0.45)
	Female	52/227 (22.9)		49.9 (41.5-55.3)		47.2 (37.6-54.0)	
Age-class, years	18-24	11/105 (10.5)	31.60 (<0.001)	55.0 (49.0-56.6)	39.33^b^ (<0.001)	47.1 (36.7-55.1)	5.05^b^ (0.28)
	25-34	19/174 (10.9)		51.9 (44.6-56.6)		47.0 (38.3-52.8)	
	35-44	10/105 (9.5)		51.9 (45.9-56.0)		46.6 (40.4-54.0)	
	45-54	20/70 (28.6)		47.8 (40.7-52.2)		48.6 (39.9-54.2)	
	55+	17/48 (35.4)		45.8 (36.3-53.5)		50.9 (41.0-56.0)	
Marital status	Never married/divorced/widowed	35/279 (12.5)	3.31 (0.07)	52.8 (45.9-56.6)	3.80^a^ (<0.001)	46.2 (36.9-53.0)	2.16^a^ (0.03)
	Married/cohabiting	42/223 (18.8)		50.5 (42.9-54.8)		48.6 (41.1-55.0)	
Schooling	Middle school or lower	21/149 (14.1)	0.14 (0.71)	50.6 (41.3-55.2)	2.22^a^ (0.03)	47.1 (37.1-54.1)	0.19^a^ (0.85)
	High school or higher	56/353 (15.9)		52.0 (44.7-56.3)		47.5 (38.4-53.9)	
HDI quartile of birth country	Low	22/121 (18.2)	9.99 (0.02)	51.8 (43.4-56.2)	5.84^b^ (0.12)	45.8 (35.9-52.6)	5.77^b^ (0.12)
	Medium	13/120 (10.8)		52.2 (43.0-55.7)		46.8 (39.3-53.9)	
	High	41/219 (18.7)		50.9 (44.1-55.6)		47.8 (38.6-54.9)	
	Very high	1/42 (2.4)		53.1 (50.7-55.8)^c^		49.4 (43.5-53.7)	
Reason for migration	Study	15/127 (11.8)	4.58 (0.10)	55.3 (49.1-57.1)	31.25^b^ (<0.001)	44.9 (36.6-52.0)	7.60^b^ (0.06)
	Work	48/257 (18.7)		49.8 (41.6-54.9)		47.2 (39.3-54.0)	
	Family	12/98 (12.2)		52.8 (44.3-56.3)		50.0 (39.1-55.9)	
	Religious	2/20 (10.0)		50.5 (43.5-54.9)		50.3 (42.0-56.4)	
Duration of residence, years	< 10	37/296 (12.5)	3.96 (0.05)	52.7 (45.5-56.6)	3.39^a^ (<0.001)	47.3 (37.9-53.6)	0.58^a^ (0.57)
	≥ 10	40/206 (19.4)		49.8 (42.9-55.2)		47.3 (39.2-54.7)	
Current smoking	Non-smoker	65/398 (16.3)	1.11 (0.29)	51.6 (43.5-55.8)	1.28^a^ (0.20)	47.1 (38.3-54.0)	0.36^a^ (0.72)
	Smoker	12/104 (11.5)		52.6 (45.2-56.4)		48.0 (38.0-54.3)	
Alcohol consumption, drinks per day	≤ 1	71/461 (15.4)	0.02 (0.90)	51.5 (43.5-55.8)	1.94^a^ (0.05)	47.5 (38.3-54.3)	1.14^a^ (0.25)
	> 1	6/41 (14.6)		53.2 (49.3-57.0)		45.9 (40.2-52.1)	
BMI, kg/m^2^	< 25	37/337 (11.0)	14.00 (<0.001)	52.2 (45.6-56.2)	3.02^a^ (<0.01)	47.1 (38.6-54.0)	0.28^a^ (0.78)
	≥ 25	40/165 (24.2)		49.8 (41.3-55.5)		47.7 (38.0-54.1)	
Medical conditions	None	27/342 (7.9)	44.01 (<0.001)	53.5 (48.1-56.6)	8.03^a^ (<0.001)	47.7 (39.7-55.0)	2.11^a^ (0.03)
	At least 1	50/160 (31.3)		45.5 (38.7-52.1)		45.7 (37.3-52.8)	

^a^: z-value of Mann–Whitney U test; ^b^: χ^2^ value of Kruskal-Wallis test.

With regard to medical conditions, about two-thirds of immigrants (68.1%) did not indicate any medical condition, while the rest reported 1–6 health problems; specifically, 24.3% reported one condition, 5.8% two conditions, and 1.8% three or more conditions. More than three-quarters of the morbidities cited fell into eight disease groups: musculoskeletal pathologies (21.6%), hypertension (13.6%), digestion problems (10.8%), respiratory pathologies (8.5%), heart disease (8.0%), anemia (7.0%), allergy (4.2%) and diabetes (2.8%). A significantly higher proportion of subjects with a single condition rated their health as poor than did those without that condition. With the exception of digestion problems, significantly lower median PCS-12 scores were also reported by participants suffering from a chronic disease than by those without, while MCS-12 median scores did not differ significantly (Table 5). The percentages of poor health status and SF-12 scores documented among subjects with anemia, allergy and diabetes were not significantly different from those without these three conditions, probably, because of the small number of people who reported these health problems.

**Table 5:** Percentage of subjects who reported poor health status and median SF-12 scores by the most frequently cited medical conditions

**Medical condition**	**Presence**	**N/total (%) of poor SRH**	**χ**^**2 **^**(p)**	**PCS-12 median score (IQR)**	**Mann–Whitney U test (p)**	**MCS-12 median score (IQR)**	**Mann–Whitney U test (p)**
Musculoskeletal pathologies	With	19/46 (41.3)	24.14 (<0.001)	38.8 (36.0-47.5)	7.14 (<0.001)	48.2 (37.7-54.0)	0.62 (0.54)
	Without	58/456 (12.7)		52.2 (45.3-56.3)		47.2 (38.3-54.1)	
Hypertension	With	12/29 (41.4)	14.01 (<0.001)	43.7 (37.8-48.3)	4.14 (<0.001)	45.4 (36.7-54.0)	0.96 (0.34)
	Without	65/473 (13.7)		52.1 (44.2-56.1)		47.5 (38.4-54.1)	
Digestion problems	With	9/23 (39.1)	8.68 (<0.01)	50.2 (38.7-53.6)	1.85 (0.06)	47.7 (38.4-53.2)	0.24 (0.81)
	Without	68/479 (14.2)		51.9 (43.9-56.0)		47.2 (38.3-54.2)	
Respiratory pathologies	With	7/18 (38.9)	6.20 (0.01)	40.4 (36.0-46.8)	4.02 (<0.001)	45.5 (34.9-53.0)	0.52 (0.60)
	Without	70/484 (14.5)		51.9 (44.2-56.1)		47.3 (38.4-54.1)	
Heart disease	With	8/17 (47.1)	11.22 (<0.001)	41.3 (35.1-44.8)	4.21 (<0.001)	40.0 (32.2-52.0)	1.72 (0.09)
	Without	69/485 (14.2)		51.9 (44.2-56.0)		47.5 (38.4-54.1)	

The strength and direction of the Spearman correlation varied among the four subjective indicators. Specifically, both PCS-12 and SRH were positively associated between themselves and negatively associated with counts of medical conditions (Table 6).

**Table 6:** Spearman’s correlation coefficients for comparisons between SF-12 scales, SRH and count of medical conditions*

	**SRH**	**PCS-12**	**MCS-12**	**Count of medical conditions**
SRH	1.00	0.49 (<0.001)	0.26 (<0.001)	-0.36 (<0.001)
PCS-12		1.00	-0.09 (0.053)	-0.37 (<0.001)
MCS-12			1.00	-0.10 (0.04)
Count of medical conditions				1.00

*: Results are reported as correlation coefficients (ρ); the associated p-values of the significance of coefficients are shown in brackets.

### Multivariate regression analysis

On multivariate logistic regression analysis, the presence of at least one medical condition proved to be the greatest determinant of both lower PCS-12 scores and poor SRH (Table 7). A similar effect was also observed with regard to BMI: overweight/obese subjects had a significantly higher probability of both poor health and low PCS-12 scores. Immigrant females had a 2.2-fold higher probability of reporting poor SRH than males, though neither the physical nor mental dimensions of HRQoL were associated with gender. At a level of statistical significance of p < 0.05, the variable “age” was not associated with any of the three outcomes of interest; however, this variable displayed a trend towards a greater probability of reporting poor health, reaching a borderline level of significance (p = 0.053). With regard to variables concerning immigration background, differences also emerged among the three outcomes: the HDI quartile of the birth country significantly influenced SRH, while the reason for migration impacted on both SF-12 scores. Thus, compared with subjects from countries with the lowest HDI quartile, those from countries with higher quartiles showed significantly lower odds of poor SRH. Conversely, neither SF-12 scale showed any association with HDI. Lower scores in the physical dimension of HRQoL were associated with immigration for work and religious reasons, while those who had migrated for religious and family reasons displayed a lower probability of lower scores in the mental dimension of HRQoL. Table 7 summarizes the results of multivariate logistic regression applied to SRH and SF-12 scales outcomes.

**Table 7:** Multivariate logistic regression analysis to predict poor SRH and less-than-median SF-12 scores

**Variable**	**Level**	**Poor SRH outcome**		**PCS-12 < 51.6 outcome**^**b**^		**MCS-12 < 47.3 outcome**^**b**^	
		**OR (95% CI)**^**a**^	**p-value**	**OR (95% CI)**^**c**^	**p-value**	**OR (95% CI)**^**c**^	**p-value**
Sex	Male	1.00 (ref)	0.010	-		-	
	Female	2.22 (1.21-4.09)					
Age-class on survey, years	18-24	1.00 (ref)	0.053	-		-	
	25-34	0.76 (0.32-1.78)					
	35-44	0.64 (0.24-1.67)					
	45-54	1.25 (0.49-3.20)					
	55+	2.47 (0.93-6.53)					
Reason for migration	Study	-		1.00 (ref)	<0.001	1.00 (ref)	0.014
	Work			2.64 (1.65-4.21)*		0.68 (0.44-1.04)	
	Family			1.28 (0.72-2.26)		0.44 (0.26-0.76)*	
	Religious			4.16 (1.50-11.57)*		0.36 (0.14-0.97)*	
HDI quartile of birth country	Low	1.00 (ref)	0.004	-		-	
	Medium	0.24 (0.10-0.58)*					
	High	0.44 (0.22-0.90)*					
	Very high	0.09 (0.01-0.72)*					
BMI, kg/m^2^	< 25	1.00 (ref)	0.005	1.00 (ref)	0.045	-	
	≥ 25	2.20 (1.27-3.82)		1.52 (1.01-2.28)			
Medical conditions	None	1.00 (ref)	<0.001	1.00 (ref)	<0.001	-	
	At least 1	3.95 (2.22-7.04)		3.45 (2.26-5.26)			

*: Significant comparisons for factors with more than two classes; ^a^: OR is considered as an increase (>1) or decrease (<1) in probability of being in poor health in each category, as compared with reference category (ref); ^b^: PCS-12 and MCS-12 are expressed in a binary manner as < median and ≥ median value; ^c^: OR is considered as an increase (>1) or decrease (<1) in probability of having a less-than-median value of SF-12 composite score in each category, as compared with reference category (ref).

Table 8 reports the results of multivariate Poisson regression applied to the count of medical conditions. Females, over-45-year-olds, long-term residents and overweight/obese people displayed higher PRs of chronic diseases.

**Table 8:** Multivariate poisson regression to predict medical conditions

**Variable**	**Level**	**PR (95% CI)**	**p-value**
Sex	Male	1.00 (ref)	<0.001
	Female	1.68 (1.26-2.24)	
Age-class at survey, years	18-24	1.00 (ref)	<0.001
	25-34	1.11 (0.67-1.82)	
	35-44	1.18 (0.68-2.03)	
	45-54	2.52 (1.49-4.26)*	
	55+	3.02 (1.77-5.16)*	
Time of residence, years	< 10	1.00 (ref)	0.01
	≥ 10	1.51 (1.11-2.06)	
BMI, kg/m^2^	< 25	1.00 (ref)	0.003
	≥ 25	1.50 (1.15-1.96)	

*: Significant comparisons for factors with more than two classes; PR: Prevalence ratio.

## Discussion

To our knowledge, this is the first study to measure HRQoL in an immigrant population in the North-West of Italy. Immigrants to Italy are an important resource because of their entrepreneurial activity, consumer spending, tax payments, participation in the national labor force and interregional equilibration of labor markets, and contribution to socio-cultural diversity. Another socially important role of immigration lies in the fact the native population is progressively aging. Indeed, the mean age of immigrants in our sample was significantly lower than that of the city’s general population, which is one of the highest in Italy (47.2 years) [60]. This is because the propensity to migrate is highest among the young [61].

The immigrants in our sample displayed moderate levels of HRQoL, and the mental component of the index was significantly lower than the physical one. Low MCS-12 scores have been found to be associated with clinical depression as well as more general measures of diminished mental health [62]. Research suggests that the two most prevalent mental health conditions among first-generation young immigrants are major depression and anxiety disorders, which can be elicited by stressful events, such as separation from the family, exposure to discrimination, traumatic events and others [63]. Indeed, in our study, those who had migrated for family reasons (family reunification) displayed a lower risk of reporting low MCS-12 scores.

### Health disparities by gender

The results of our study highlight the presence of health inequalities within the immigrant population. First of all, we documented the presence of a gender difference, in that the health status of women proved to be poorer and displayed a higher prevalence of medical conditions than that of men, even after adjustment. This phenomenon has been amply demonstrated in both developed [64,65] and developing countries [66,67]. Similarly, studies on migrant health have found poorer health status [68] and a higher prevalence of chronic diseases [57] among female immigrants than among males. This seems paradoxical (“gender paradox”) as the life expectancy of men is shorter in almost all countries [69]. This gender divide is probably the result of the interplay between biological and social factors [18]. It has, for example, been suggested that women have a more acute perception of their medical problems, especially minor problems, than men do and, consequently, that they are more likely to report them [70]. However, the gender difference in self-assessed health indexes has been shown to be complex and to vary across age, social context and, obviously, morbidity background [71]. The gender divide may be particularly marked in migrant populations, as it is well known that immigrant women are particularly vulnerable to health problems and often have less access to prevention and healthcare [72].

### Impact of reason for migration and duration of residence on subjective health indicators

In our study, subjects who had migrated for work reasons had a higher probability of reporting lower physical HRQoL, which is in contrast with the notion that those who migrate in search of work are generally young and healthy [73]. However, the health of labor migrants is often vulnerable as a result of various risk factors, such as lack of adequate health insurance, poverty and uncertain legal status [74]. Moreover, immigrants are often employed at the lower end of the labor market [75] even if they have a high level of education, a situation that may give rise to education-to-job mismatching. In this regard, it has been shown that overqualified immigrants, especially those of recent arrival, are at high risk of work-related injury [76]. This phenomenon could be particularly pronounced in Southern Europe, where there is a greater demand for unskilled and low-skilled migrants than for highly skilled ones. The most common problems associated with this fact include difficulties in social integration and uncertain long-term job prospects among low-skilled immigrants. In Italy, low- and unskilled jobs are often unregulated, which means that it is difficult for such immigrants to find regular employment [77]. Low-skilled jobs, in turn, often involve working in poor environmental conditions or without adequate safety [78]. Indeed, according to the Italian Workers Compensation Authority, immigrants are often engaged in particularly risky activities, work longer shifts and lack adequate vocational training, which results in a higher incidence of professional accidents [79]. Immigrant workers in Italy also display relatively high rates of work-related musculoskeletal diseases [80], which were the most frequently reported disorders in our study.

We found that migrants for religious reasons had a higher risk of poor physical HRQoL; by contrast, mental HRQoL scores were significantly higher in these subjects than in other groups. Indeed, research has often revealed a salutary effect of religious involvement, especially on mental health [81-83]. On the other hand, some research has underscored the possibility of a spurious association between health and religion [84]. Indeed, Sloan et al. [85] noted a weak and inconsistent association between health outcomes and religious practices. Moreover, Shmueli [86] documented an adverse effect of religiosity and spirituality on health, as measured by the SF-36 and visual analogue rating scale, thus confirming the ambiguity of the effect of religion on health. On the other hand, Franzini et al. [83] found a positive association between religiosity and a 5-point SRH scale, but no association between SF-12 scales and religious involvement among Mexicans in the United States.

Subjects who had migrated for family reasons showed higher MCS-12 scores on multivariate regression. This group comprised migrants for the purpose of family reunification and consisted of spouses, parents and children of immigrants as well as those seeking reunification with an Italian citizen. The better mental health of such subjects could be explained by the absence of exposure to pre-migration health risks, the relative ease of the migration process and social support from relatives who have already settled in the host country [87].

Long-term immigrants have been found to be at higher risk of chronic conditions. Dunn and Dyck [57] found a significant difference in reported health conditions according to the duration of residence, non-recent immigrants (10 or more years) being more likely to report at least one health condition (OR = 1.74, p < 0.001). Another Canadian study found that the time since immigration impacted differently on men’s and women’s health: long-term immigrant women were significantly more likely to report poor health than recent ones, while no significant difference was seen among immigrant men [88]. Similarly, we found a higher prevalence of medical conditions among both long-term immigrants and women.

### Health and country of birth

Our study highlights the importance of pre-migration factors, particularly that of the immigrants’ birth countries; indeed, people from less developed societies were more inclined to report poor health status. Similar results also emerged from a Canadian study in which immigrants from countries with lower HDI values reported poor SRH more frequently than those from countries with higher HDI values [37]. Clearly, the HDI variable was used as a proxy measure. However, we believe that this broad indicator might be useful in migrant studies, since it is well documented that the distribution of material, capital, human and other key resources is an important predictor of a population’s average levels of health, income and education (which are the three dimensions of the HDI) [89]. It is therefore plausible that migrants bring their “health luggage” with them to a host country. For example, lower SF-12 scores have been reported in the general Iranian population (HDI = 0.707) [58,90] than in the general populations of nine developed European countries (HDI ranges from 0.861 to 0.943) [54,58]. However, this finding should be interpreted with caution for at least three reasons. Firstly, as the most important reason for migration is to improve quality of life [41], the health status of immigrants in a host country may not precisely reflect the baseline pre-migration level. Secondly, observed differences in SRH may be due to cross-cultural differences in health perception [91]. Finally, in accordance with the self-selection hypothesis, immigrants might initially be in better health than their compatriots who do not migrate, but subsequently their health might deteriorate over time, probably as a result of a combination of environmental and behavioral changes [5,92].

### Negative influence of overweight and obesity on SRH and physical HRQoL

Our results showed that being overweight or obese is a risk factor for poor health reporting as well as for lower scores of physical HRQoL; this is in line with previous studies [93-96]. In particular, Huisingh-Scheetz et al. [96] found an inverse independent association between PCS-12 and BMI and no association between MCS-12 and BMI among different racial and ethnic subgroups. Notably, the degree of this association among whites, blacks and Hispanics was quite similar, which indicates that programs to reduce obesity may yield an equal improvement in the physical dimension of HRQoL in all ethnic subgroups.

### Different ability of subjective health indicators to predict health disparities

Finally, it is important to note that, while the different subjective health indicators are able to reveal inequalities within immigrant populations, they do so in different ways. Thus, morbidity and BMI were similarly associated with PCS-12 and SRH. Indeed, both PCS-12 and SRH displayed good ability to distinguish between immigrants with a specific health problem and those without that problem, as well as between overweight/obese and normal-weight subjects. Self-reported morbidity, in turn, can be used to predict quality of life and should be regarded as a reliable measure for risk adjustment in immigrant populations. On the other hand, only SRH was able to predict disparities by sex, HDI and BMI. By contrast, both SF-12 scores were associated with the reasons for immigration. Kaplan et al. [97] also found a moderate level of correlation between HRQoL and SRH; in their study, SRH predicted mortality better than HRQoL. These authors also found that, when there was overlapping between the two indicators, each of them provided new information. The results of recently published research conducted by Delpierre et al. [35] indicated that, in measuring inequalities, SRH may downplay socioeconomic differences, while HRQoL highlights them.

### Study limitations

Our study is not without limitations. First of all, we used non-probabilistic sampling techniques, which are subject to some biases, selection bias *in primis*; the results cannot therefore be generalized [98]. However, even if the use of random techniques had been possible, the sampling frame would have been incomplete, as about 40,000 foreigners are granted Italian citizenship each year and disappear from official statistics. Specifically, citizenship can be applied for by long-term immigrants after 10 years of residence or after 2 years of marriage to an Italian citizen [99]. However, chain-referral methods can have some advantages; in particular, they can enable hard-to-involve populations to be reached through their social networks [100]. To mitigate the weaknesses of this sampling method, we started the snowball chain from people belonging to different social groups.

Second, the subjects enrolled in our study were sometimes inhomogeneous in terms of age and sex, since individual communities of immigrants display different patterns. For example, 85% of Ukrainian immigrants are women, while 82% of migrants from Senegal are men [2,60]. Such inhomogeneity also applies to the numbers of participants born in each country; indeed, some foreign communities, such as Chinese, were underrepresented. On the other hand, the majority of immigrants to Genoa are from countries with high HDI, while those from very high HDI societies account for less than 10% [2,58]. The use of HDI groupings can mitigate this limitation. However, our sample was age-representative, as the mean age of the study population reflected that of the whole foreign adult population of Genoa (37.6 years for males and 39.4 years for females) [60]. Similarly, the sample was representative of civil status [101].

Thirdly, we only enrolled subjects who had a sufficient knowledge of the Italian language, which could constitute a barrier for the most recent immigrants. Indeed, 3% of subjects were unable to fill in the questionnaire and were excluded. However, the German version of SF-12 has been successfully used among different ethnic groups in Germany [41].

Fourthly, our questionnaire did not contain some important items – such as housing, employment and working conditions, utilization of health services, etc. – that could affect health outcomes. The same applies to the issue of legal presence in Italy. A previous version of the questionnaire contained an item regarding the legality of residence in Italy. However, in our pilot study, several subjects (7 out of 30) omitted this question, even though they were assured that the questionnaire was anonymous. Therefore, in order to encourage openness and honesty in the answers, this question was deleted. Moreover, Pikhart et al. [36] did not find a statistically significant difference in terms of poor SRH reporting between legal and illegal immigrants in the Czech Republic.

## Conclusions

Our study points out the presence of health inequalities within the immigrant population in Genoa. Subjective health indicators should be viewed as important tools for public health policies, as they are valid, useful and relatively cheap, and can help to identify vulnerable population subgroups and to monitor progress in implementing migrant-sensitive healthcare programs.

As immigration to Italy has increased significantly [2], immigrant health should be one of the most important public health issues. Moreover, as the immigrant population in Italy is very heterogeneous, public health authorities should ensure the development and implementation of multi-sectorial strategies to reduce health inequalities not only between immigrants and natives, but also among different immigrant groups, and to promote health among immigrants through clearly designed information. In this regard, the World Health Organization (WHO) has identified four principles to be embodied in public health policy: (1) ensuring migrants’ health rights; (2) reducing excess mortality and morbidity; (3) avoiding disparities in health status and access to healthcare services, and (4) minimizing the negative impact of the migration process on immigrant health [73].

## Abbreviations

BMI: Body mass index; CI: Confidence interval; GNI: Gross national income; HDI: Human development index; HRQoL: Health-related quality of life; IQR: Interquartile range; ISTAT: Italian national institute of statistics; MCS: Mental component summary; OR: Odds ratio; PCS: Physical component summary; PR: Prevalence ratio; SF-12: Short form-12; SRH: Self-rated health; SD: Standard deviation; UN: United nations; WHO: World health organization.

## Competing interests

The authors declare that they have no competing interests.

## Authors’ contributions

RG supervised the research; AD, EBR and RG designed the study and coordinated the research; AD optimized the informatics database; AD, DA, DP, SA and AS performed the statistical analyses and evaluated the results; AD, DA and DP wrote the manuscript; AD, DP, VP and SA collected data and carried out the first local quality control; all authors revised the manuscript and contributed to improving the paper; all authors read and approved the final manuscript.

## Pre-publication history

The pre-publication history for this paper can be accessed here:

http://www.biomedcentral.com/1471-2458/13/1006/prepub
